# Army Public School Terrorist Attack - Perceptions and Experiences of school children of Karachi

**DOI:** 10.12669/pjms.38.7.5596

**Published:** 2022

**Authors:** Mehjabeen Musharraf, Lubna Ansari Baig, Zarrukh Ali Baig

**Affiliations:** 1 Mehjabeen Musharraf, MSc, MSPH. Senior lecturer, APPNA Institute of Public Health, Jinnah Sindh Medical University, Karachi, Pakistan; 2 Lubna Ansari Baig, FCPS, PhD. Chairperson, APPNA Institute of Public Health, Jinnah Sindh Medical University, Karachi, Pakistan; 3 Zarrukh Ali Baig, MBBS. Resident Surgery, University of Saskatoon, Saskatchewan, Canada

**Keywords:** APS attack, Effects of terrorism on school children, indirect exposure of terrorism, Media and terrorism, Experiences and perceptions of school children after a terrorist attack

## Abstract

**Objectives:**

To explore the experiences and perceptions of school children of Karachi after the Army Public School (APS) attack.

**Methods:**

It was a qualitative transcendental phenomenological study. Data collection started nine months after the attack, in September 2015, and continued till November 2019. Study participants were school children from Army, Government, and Private schools. The sampling strategy was convenience. Data collection of 53 students was done by focus group discussions and in depth interviews. Data analysis was performed using the phenomenological analytical techniques of Colaizzi.

**Results:**

Inductive analysis of the qualitative data gave rise to three themes - The journey beyond fear, Response of parents and schools and Role of media.

**Conclusion:**

The study concluded that the APS attack was the source of emotional distress and fear for the school children of Karachi as they personalized the event due to the nature of the attack. Immediately after the incident, they were in anger, grief, and fear, which altered their daily life activities and caused apprehensions in socializing and attending school. However, later they became highly motivated to study and gained courage. This motivation is revenge from terrorists as they wanted to keep children away from schools.

## INTRODUCTION

South Asia is the region most affected by terrorism in 2019 though the incidents of terrorism have decreased in Afghanistan, Pakistan and India.^1^ Pakistan has been the victim of terrorism for many years, and ranks seventh among the ten countries most affected by terrorism.^1^ On December 16, 2014, the Peshawar city of Pakistan faced a terrorist attack that targeted school children. Seven armed terrorists equipped with grenades and automatic rifles attacked the Army Public School (APS) managed by the Pakistani Army’s Army Public Schools & Colleges System.^2^ It claimed the lives of 157 people, including school children, the principal, and other staff members.^2^ Targeting children in schools is attack on education for keeping them away from schools. This is done because education develops the skill of critical thinking, which makes young people question the concepts of hatred and decrease the violence and terrorism from societies. Therefore, it is essential to make the school environment safe and secure and make education more accessible.^3^

After six months of this attack, PTSD was evaluated in APS attack surviving children, 10 to 18 years of age. The study reported PTSD in 75.2% of children and 50 % PTSD positive school children showed functional impairment for school work, fun and hobbies, saying prayers, friendship, family relation, general happiness and doing chores.^4^ Children are more likely to experience the adverse effects of terrorism as compared to adults and have a more challenging time returning to everyday life.^5^ Also, the indirectly exposed children who got the exposure through media coverage of terrorist attacks, may exhibit the clinically significant psychological and emotional effects.^6^ This childhood exposure (direct/indirect) of terrorism increases the chances of developing the emotional symptoms if the episode is repeated in adult life.^5^

Considerable work is done in developed countries to explore the impact of terrorism in directly and indirectly exposed populations^6^,^7^ however, the findings do not apply to Pakistan due to the marked differences in the socio-cultural, geographical and political aspects. Hence, it was imperative to do a qualitative study in this region to explore the perceptions and experiences of the indirectly exposed children after APS Attack. The psychological effects in the communities that experience terrorism decrease with time provided no future threats but in a country like Pakistan, which continuously faces terrorism in more unpredictable form each passing day, it is less likely to anticipate a decrease.

The concept given by Joshi & O’donnell was used as a theory in these which states that adolescents keep the feelings of trauma to themselves and pretend as if they are fine. This behavior makes them more susceptible to the psychological distress.^8^ The study, therefore, employed qualitative methods so that the children can open up and talk about their hidden fears and emotions. The current study aimed to explore the experiences of school children of Karachi after the APS attack in terms of their feelings and impact on their lives, considering the high frequency of terrorism in Karachi, which serves as a continuous reminder to them.

## METHODS

The study was planned, executed and analyzed based on COREQ (Consolidated Criteria for Reporting Qualitative Studies) guidelines. A qualitative transcendental phenomenological approach yielded detailed insights into the lived experiences of Karachi School children after the APS attack. This approach is concerned more with participants’ experiences and significantly less with the researchers’ interpretations.^9^ Therefore, the authors penned down their views about the topic to ensure rigour. The first author is currently a public health practitioner, but in 2014 when the incident occurred, she was working as a middle school teacher. She observed that children became very restless as they could not go outside their classrooms even at lunchtime and the physical activity lessons. They would show aggressive behavior in classrooms, and maintaining discipline in the classroom became very difficult for the teachers. The second author is a medical educationist, public health leader, and academician. According to her, parents were worried regarding the safety and security of the schools after the APS attack. Being an academician, she wanted to explore how terrorism affects children’s mental health and what measures can mitigate the repercussions. The third author is a medical Doctor currently working in Canada. At the time of incidence, he was a medical student and compiling a video on underprivileged children for HELP a local NGO. He was deeply shaken by the incident and was concerned about the effect it may have on children and wanted to identify measures that can mitigate its effect on their mental health status.

Participants were school children, 13-15 years of age, in grades 9 - 11 from Matriculation and O level education systems. Sampling was convenience, and participants were from the army, public and private schools. Data collection started nine months after the attack, in September 2015, and continued till November 2019. Data saturation was achieved after conducting six focus group discussions (FGDs) and five in-depth interviews (IDIs). Permission was sought from the Institutional Review Board of Jinnah Sindh Medical University (JSMU/IRB/2015/-13) and the school authorities. The principals of the selected schools were briefed about the research, and they suggested suitable dates, time and participants and signed the consent form after getting permission from parents. The participants signed the assent form. The duration of interviews was 30 to 40 minutes and were audio and videotaped, and later transcribed verbatim. Dependability was maintained by listening to the random videos and audios thrice to check the accuracy and completion. The primary researcher was accompanied by a note-taker who managed the recordings and took notes of the whole discussion for data collection. An interview guide was used with these main questions: Describe your feelings about the APS attack (Recall that time and present feelings). How did the APS attack affect your life (Recall that time and present feelings)? Describe your all experiences so far related to the attack?

To ensure credibility, participants were asked to describe their experience of living after this incident without influencing their description. Phenomenological analytical techniques of Colaizzi^10^ were used for analyzing the data. The first two transcripts and note-taker’s notes were read separately; significant statements were identified, and meaningful statements were formulated and themes were generated. All of the three researchers finalized the themes after discussion with consensus. Data were later deductively analyzed for the identified themes and the responses of FGDs and IDIs were triangulated.

## RESULTS

Fifty-three students participated with 48 (91%) in FGDs and 5 (9%) in IDIs with the median age of 15 years five months. Twenty-seven (51%) were males, and 26 (49%) were females. 34 (64%) were from Grades-9 and 10, with 11(21%) from grade 10, 8 (15%) from A1. Out of six schools, four were private, one was the government, and one army. (Table-I)

**Table-I:** Demographic characteristics of study participants.

Characteristic	Averages
***Age (years)***	
Range	14-17
Median	15.5

***Characteristics***	***Frequency (%)***

***Gender***	
Male	27 (51)
Female	26 (49)
***Education level***	
9^th^/O1	34(64)
10^th^/O2	11(21)
A-1	8(15)
***School Type***	
Govt.	1 (16)
Private	4 (67)
Army	1 (16)

### Theme-1 The journey beyond fear:

Many of the participants said that after the attack, they got terrified and were afraid that it could be repeated with them. The intense feelings of fear lasted for three months, and later they became fearless, but the thought of the attack still fill their eyes with tears.

***“Every morning, we had this thought that what if it happens in our school, so every step towards school was like a fearful step, but now we are strong and study with more passion” (15 years old from a private school)***

***“We were horrified and fearful, but still, we did not lose hope and went school the next day” (16 years old student from a private school)***

***’At that time, we were in so much pain and anger that if we found someone from those people, we would crush them. We want to eliminate them from roots’’ (15 years old from the Government school)***

According to the participants, perpetrators must be punished and hanged to death. The comments of the school children were very harsh when it came to punishing the culprits of the APS attack. One of the participants urged to burn the culprits in front of the public.

Under this theme, the dominant most idea was that the APS attack has made the students more motivated to study, and they have started working even harder. They consider this motivation the biggest revenge from those terrorists because attacking the school was to keep the children away from school and education. They said that school children gained maturity and started discussing politics after the incident; they felt that terrorists had left them more confident and stronger.

### Fighting with terrorists was discussed in two ways

pen, meaning by getting the best education and failing their agenda, and joining the army.

“We can fight with a pen against terrorism” (16 years old from Government school).

“We have a pen; they have a sword. The pen is stronger than the sword” (16 years old from the school run by Army)

### Theme-2 response of parents and schools:

Participants said that parents were more fearful than children. They restricted their socialization and were reluctant to send them to school.

***“My parents were miserable because they were feeling the pain of those who lost their children, but we (children) kept our emotions to ourselves; otherwise, our parents would not have sent us to school for some period. (16 years old from a private school)***.

A child from the private school shared that after this incident, the whole family would sit together, and parents counsel him by saying,

***“They(terrorists) may keep doing this, but you should not be afraid of it; if you get afraid, they will accomplish what they want, but we will not make it happen”***.

Almost all of the participants said that schools increased security after the APS attack. Some schools also received threats after the incident. For a few days, the school management kept the class windows closed to give the vacant look of the school from outside. In-home time, the administration would not allow students to leave the school alone. The next day after the attack, principals gave a motivational speech and counselled the students. Schools did special prayers for departed souls and families and hoisted down the flags. Everyone was sad that day; teachers counselled them and taught them to be strong and brave. After this incident, schools have stopped taking them to field visits. For some time, they did not have their physical training sessions and were not even allowed to go out of the class during lunchtime. Children got absent for a few days due to the fear but later became regular because they were satisfied with the security measures taken by schools. A student with an army background mentioned that absenteeism was decreased in his school, and the students who were habitual of getting absent from school before the attack became regular afterwards.

### Theme-3 role of media

They said that media is insensitive and do not care about people with a sensitive heart. The news channels and social media shared the videos of the APS attack; such coverage should have been avoided because those scenes made them cry a lot. School children followed the news of the APS attack on television and social media.

***“They should discourage media as in the race of ratings; news channels show all negativity over and over. They were showing barbarism; it was something that I was unable to stop my tears (coverage of APS attack)”.15 years old from a private school)***.

“***The news channels cared about their ratings; they grabbed videos from places and showed them on TV to get their ratings, but they do not think about the people who have lost someone close to them”(15 years old from a private school) On the contrary, some students said that it is good that media alarms and prepare for any situation as it is crucial to be updated and prepared.***

## DISCUSSION

School and education are targeted with the purpose of instilling fear^11^ and this idea is validated by our study as the dominant most response of school children was feeling of insecurity for themselves and siblings and fear of going to school due to the apprehensions of similar future attacks. This finding is similar to the study done in Khyber Pakhtunkhwa according to which 93% of the secondary school children said that terrorism cause fear in them.^12^ The study however could not elaborate on the manifestation of fear as it was a quantitative study. The identification of fear in children emphasizes the need to formulate contextually relevant guidelines for the schools and parents in case of terrorism to reduce the fear and apprehensions in children. Though the incident occurred in Peshawar, the school children of Karachi also reported the fear of getting the incident repeated with them. This is because they were personalizing the event and considering themselves a potential victim.

Most of the effects settled within three months of the attack, but some persisted. One of them was a decrease in extracurricular activities, field trips in schools, and social gatherings of children. Field trips provide experiential learning opportunity and help teachers build knowledge skill and attitude about various concepts. These trips provide rich experiences to children and are essential for the all-developmental domains, including social-emotional development.^13^-^15^

The study revealed that the children found themselves more inclined towards education after the APS attack. Immediately after the attack, they were scared and fearful but later showed positive feelings of motivation towards acquiring education. As most studies on terrorist attacks and their impact were conducted in developed countries with different socio-political structures, motivation to study as the mean of resilience is not evident from literature;^7^,^16^,^17^ however, this is the most pervasive idea in this study. The song made in the memory of APS martyrs might have helped them turning their fears into motivation to study.

***“Mujhai maan us sai badhla lainai jana hay mujhai dhushman kai bachho ko parhana hay”(Mom I have to go to take revenge from him by educating his children)***

***Mujhai jana para hay per maira bhai karai ga ab, may jithna na parha maira bhai parhai ga ab (I have died, but my brother will do whatever I could not have done and studied)***

The way media has affected children’s perception highlights its importance in shaping up their behaviour and responses. In the case of the APS attack, media has played a significant positive role in propagating motivation to study and has given them strength. Nevertheless, unfortunately, the media has also played a negative role by showing heart throbbing pictures of children and the place of the incident. This finding is consistent with another study on terrorism, indicating that the stress reactions subside in the population not directly exposed to terrorism. However, the media serves as a continuous reminder due to which stress symptoms remain persistent.^18^-^20^ Public health professionals and media (social, print, electronic) can closely work together to mitigate the after-effects of any disaster.^21^-^23^

This study has filled the knowledge gap by identifying the perceptions and experiences of schoolchildren of Karachi about the APS attack, but what permanent effects such attacks will bring to their cognitive, emotional, and behavioral function as adults is still a question mark. Recall bias is a limitation of the study as the study started nine months post-APS attack.

## CONCLUSION

The findings of this study imply that the APS attack brought fear and emotional distress in school children of Karachi as they personalized the event due to the nature of the attack. Motivation to study as revenge to terrorists was a positive effect that helped them cope with the immediate feelings of insecurity of going to school. Counseling by parents and schools helped the indirectly exposed children coming out of grief and fear after the terrorist attack. Therefore, there is a need to train teachers and parents to reduce the post terrorism stress manifestations significantly. Media is a powerful tool and must be positively utilized to channel people’s attitudes and minds towards constructiveness and optimism. It can mould the actions and attitudes of people in a way that can bring peace, tolerance, and balance to society.

### Authors’ Contribution:

**MM:** Conceived the idea, identified the participants, conducted in-depth interviews and focus group discussions, performed analysis and wrote the first draft. She was responsible for the overall accuracy of the work.

**LB** Supervised data collection and was the second person analyzing transcripts, she edited the second draft.

**ZB:** was part of the analysis team, did literature search, worked on editing and participated in manuscript writing.

## References

[1] (2020). Vision of Humanity Top 10 Terrorist Countries:Global Terrorism Index 2020.

[2] Mufti KA, Mufti AA, Bresnahan M The Army Public School Massacre in Peshawar, Pakistan An International Perspective on Disasters and Children's. Mental Health.

[3] reliefweb Safe Schools:The Hidden Crisis - A Framework for Action to Deliver Safe, Non-violent, Inclusive and Effective Learning Environments. 2018 Jan 20, 2022].

[4] Khan AO, Ullah, Nawaz K (2018). Post traumatic stress disorder among school children of Army Public School Peshawar after Six month of terrorists attack. Pak J Med Sci.

[5] Prieto S, Sanz J, García V, María P, Fausor R, Morán N (2021). Growing Up with Terrorism:The Age at Which a Terrorist Attack Was Suffered and Emotional Disorders in Adulthood. Front Psychol.

[6] Leiner M, Peinado J, Villanos Maria TM, Lopez I, Uribe R, Pathak I (2016). Mental and Emotional Health of Children Exposed to News Media of Threats and Acts of Terrorism:The Cumulative and Pervasive Effects. Front Pediatr.

[7] Durodie B, Wainwright D (2019). Terrorism and post-traumatic stress disorder:a historical review. Lancet Psychiat.

[8] Joshi PT, O'donnell DA (2003). Consequences of child exposure to war and terrorism. Clin Child Fam Psychol Rev.

[9] Creswell JW (2014). Qualitative, quantitative and mixed metho ds approaches. Sage.

[10] Wirihana L, Welch A, Williamson M, Christensen M, Bakon S, Craft J (2018). Using Colaizzi's method of data analysis to explore the experiences of nurse academics teaching on satellite campuses. Nurse Res.

[11] Petkova EP, Martinez S, Schlegelmilch J, Redlener I (2017). Schools and Terrorism:Global Trends, Impacts, and Lessons for Resilience Stud. Confl. Terror.

[12] Bilal M, Inamullah HM, Irshadullah HM (2016). Effects of terrorism on secondary school students in Khyber Pakhtunkhwa. Dialogue.

[13] Kizıltas E, Sak R (2018). Integrating field-trip activities with other activities in the preschool curriculum:its effects on the preschoolers'social–emotional skills. Int J Child Care Educ Policy.

[14] Young T (2021). Unpacking the Field Trip Creating Meaningful Museum Experiences for K–12 Audiences. How to Connect with Teachers and Engage Students.

[15] Achen RM, Warren C, Fazzari A, Jorich H, Thorne K (2019). Evaluating Graduate Student Out-of-Class Learning:The Professional Field Trip. Int J Teach Learn Higher Educ.

[16] Grenon M, Consigny M, Lemey C, Simson, J P, Coulon N (2019). Impact of a Terrorist Attack on the Mental Health of Directly Exposed French Adolescents:Study Protocol for the First Step of the AVAL Cohort Study. Front Psychiatry.

[17] Sirin SR, Choi E, Tugberk C (2021). The Impact of 9/11 and the War on Terror on Arab and Muslim Children and Families. Curr Psychiatry Rep.

[18] Holman EA, Garfin DR, Lubens P, Silver RC (2019). Media Exposure to Collective Trauma, Mental Health, and Functioning:Does It Matter What You See?. Clin Psychol Sci.

[19] Holman EA, Garfin DR, Silver RC (2014). Media's role in broadcasting acute stress following the Boston Marathon bombings. PNAS.

[20] Pfefferbaum B, Tucker P, Pfefferbaum RL, Nelson SD, Nitiéma P, Newman E (2018). Media Effects in Youth Exposed to Terrorist Incidents:a Historical Perspective. Curr Psychiatry Rep.

[21] Poddar S, Mondal M, Ghosh S (2007). A Survey on Disaster:Understanding the After-effects of Super-cyclone Amphan and Helping Hand of Social Media. arXiv preprint arXiv.

[22] Parida D, Moses S, Rahaman KR (2021). Analysing media framing of cyclone Amphan:Implications for risk communication and disaster preparedness. Int J Disaster Risk Reduct.

[23] Jetter M (2017). The effect of media attention on terrorism. J Public Econ.

